# Skeletal Muscle Aging Is a Significant Contributor to Systemic Aging

**DOI:** 10.3390/biom16101527

**Published:** 2026-10-20

**Authors:** Mei-Mei Li, Xinwei Liu, Yixuan Li, Han Li, Yuhao Wang, Kangli Qiu, Wen Kong

**Affiliations:** 1 Department of Endocrinology, Union Hospital, Tongji Medical College, Huazhong University of Science and Technology, Wuhan 430022, China; m202476270@hust.edu.cn (M.-M.L.); m202376123@hust.edu.cn (X.L.); m202275836@hust.edu.cn (Y.L.); d202482322@hust.edu.cn (H.L.); u201710310@hust.edu.cn (Y.W.); connie_qiu@hust.edu.cn (K.Q.); 2 Diabetes and Metabolic Disease Clinical Research Center of Hubei Province, Wuhan 430022, China; 3 Hubei Key Laboratory of Metabolic Abnormalities and Vascular Aging, Huazhong University of Science and Technology, Wuhan 430022, China; 4 Hubei Branch of National Center for Clinical Medical Research of Metabolic Diseases, Wuhan 430022, China

**Keywords:** aging, skeletal muscle, regeneration, myokine, age-associated disease

## Abstract

Skeletal muscle aging is a conserved biological process characterized by progressive declines in muscle mass, strength, regenerative capacity, and metabolic adaptability, contributing to impaired mobility, frailty and age-related diseases. Beyond its canonical role in locomotion, skeletal muscle functions as an endocrine and metabolic organ that actively regulates organismal aging through myokines, metabolites, extracellular vesicles, thermogenic activity, and dynamic communication with distant organs. In this review, we summarize the intrinsic mechanisms driving skeletal muscle aging, including protein turnover imbalance and anabolic resistance, myosteatosis, denervation and motor unit loss, and cellular composition remodeling. We also discuss how aging skeletal muscle influences systemic aging through bidirectional interactions with metabolic organs, brain, cardiovascular system, and bone via endocrine signaling and interorgan communication. Finally, we highlight emerging interventions targeting skeletal muscle aging, including exercise, nutritional strategies, pharmacological approaches, and other novel therapies. Understanding skeletal muscle as both a target and regulator of systemic aging provides a framework for developing strategies to promote healthy aging and reduce the burden of age-associated diseases.

## 1. Introduction

Aging is a universal and multifactorial process that impairs tissue and organ function and increases susceptibility to major diseases. While not classified as a disease itself, aging is a key risk factor for life-threatening conditions [1]. As the burden of age-related diseases grows in an aging population, research into aging mechanisms deepens, aiming to better understand the process. Although tissue-specific mechanisms are traditionally investigated, increasing evidence suggests that organismal aging emerges from dynamic interactions among different systems rather than independent deterioration of organs. A recent review expands canonical aging hallmarks from 12 [2] to 14 [3] and emphasizes interactions across cell types and organs. Therefore, identifying tissues that actively regulate the aging process represents an important challenge.

Skeletal muscle constitutes 30–50% of human body mass and is the largest metabolic reservoir, responsible for glucose homeostasis and energy metabolism. It also acts as an endocrine organ, secreting myokines, metabolites, and extracellular vesicles. In addition, it is a highly regenerative tissue that responds to exercise, nutrition, and stress. However, age brings degenerative declines in muscle mass and strength, a common, complex geriatric condition clinically defined as sarcopenia [4]. Quantitative data demonstrate that muscle strength increases to peak in early adult life, is maintained through to midlife, and declines from midlife onwards [5]. Males were on average stronger than females from adolescence. Median grip strength peaks at approximately 51 kg in men between the ages of 29 and 39, compared to 31 kg in females between the ages of 26 and 42, followed by progressive decline, with the grip strength decreasing to approximately one quarter by the age of 80 [5]. Additionally, longitudinal primate data show that the magnitude of age-related muscle mass declines, with vastus lateralis muscle mass reduced by approximately 39% in old compared with young animals [6]. Moreover, debilitating diseases resulting from muscle loss in aging significantly impair quality of life and limit lifespan [7]. Studies show muscle depletion is implicated with shorter life expectancy, independent of body mass index, especially among those with cancer [8,9,10,11,12]. These unique characteristics position skeletal muscle not only as a tissue vulnerable to aging, but also as a potential regulator capable of shaping systemic aging progress.

Recent reviews have greatly advanced our understanding of skeletal muscle aging. Clinical perspectives have established sarcopenia as an important geriatric syndrome, summarizing diagnostic criteria and management strategies [13]. Mechanistic studies have comprehensively described the pathology and etiology in aging muscle, including fiber-type remodeling, satellite cell exhaustion, inflammaging, proteostasis and mitochondrial impairment, neuromuscular junction degeneration, and interventions [14]. More recent studies highlighted myokine-mediated communication between skeletal muscle and distant organs [15,16]. However, these studies have primarily focused on either muscle-intrinsic degeneration or specific signaling pathways. An integrated mechanistic framework explaining how skeletal muscle contributes to systemic aging, and how systemic aging environments influence muscle deterioration, remains incomplete.

In this review, we propose that skeletal muscle plays a central role in the aging network, functioning as a target, mediator, and amplifier of systemic aging. Specifically, we first discuss the conserved changes in aging muscle, including protein turnover imbalance and anabolic resistance, myosteatosis, denervation and motor unit loss, and cellular composition remodeling. Secondly, we examine how aging muscle communicates with other organs through endocrine and metabolic pathways, emphasizing bidirectional interactions with the nervous system, bone and metabolic organs. Finally, we evaluate current therapeutic strategies and discuss their translational challenges, including limitations of animal models and the need for human validation.

## 2. Conserved Changes in Aging Skeletal Muscle

**Figure 1:**
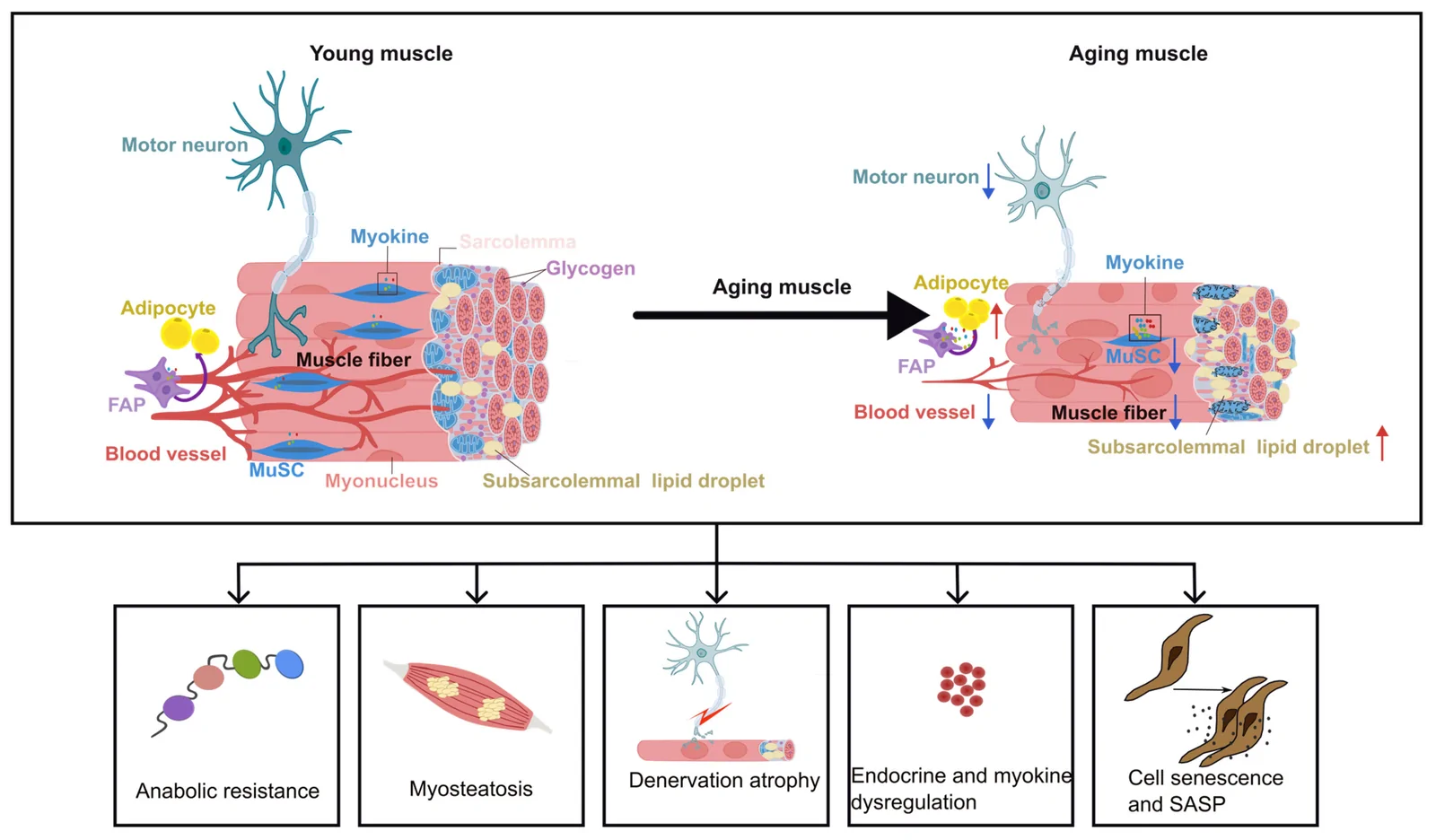
Changes in aging skeletal muscle and main impacts on organismal metabolic homeostasis and aging. This illustration depicts the microanatomy of skeletal muscle, highlighting the influences of aging muscle on the niche and systemic homeostasis. In aging skeletal muscle, anabolic resistance blunts muscle protein synthesis, and progressive denervation and motor unit loss drive functional decline; myosteatosis, adipose infiltration of muscle, further compromises muscle quality and metabolic health. The interstitial space contains various cells, including muscle stem cells (MuSCs), fibro-adipogenic progenitors (FAPs), adipocytes, immune cells, and endothelial cells, which interact with muscle and systemic homeostasis via myokines, which change significantly with aging. The synergistic action of myokines and the senescence-associated secretory phenotype promotes systemic aging. Blue downward arrows indicate decreased physiological function or reduced abundance, whereas red upward arrows indicate functional abnormalities or increased abundance.

Aging muscle experiences fiber atrophy, lipid deposition, denervation, and impaired metabolic flexibility. Skeletal muscle maintains mobility and posture via contraction, manages glucose, lipid, and amino acid metabolism for energy and exerts anti-inflammatory and immunomodulatory effects via myokines [17]. Below, we review the muscle’s functional and cellular composition, its aging-related changes, and their conservation across species, as shown in Figure 1.

Sarcopenia, age-related loss of muscle mass and function, emerges in midlife and accelerates thereafter, contributing to frailty, disability and mortality [5,6]. In aging models, like Drosophila and mice, skeletal muscle altered significantly with age [18,19,20,21]. Age-related changes in muscle may initiate aging in other tissues [22].

### 2.1. Impaired Proteostasis, Metabolic Plasticity, and Endocrine Regulation

Muscle mass is governed by the dynamic balance between muscle protein synthesis (MPS) and breakdown (MPB). Nutrition and physical activity drive the anabolic response [23,24], primarily via mTORC1 signaling [25], stimulating MPS and inhibiting MPB to maintain mass. Muscle is highly adaptable, adjusting fiber composition and size in response to nutrition and exercise, but this adaptability is eroded with aging, affecting anabolic function and promoting atrophy [26]. Aging contributes to this adaptive anabolic response and the imbalance, leading to anabolic resistance, a blunted response to anabolic stimuli. Meta-analyses confirm that the impaired anabolic responsiveness represents a fundamental mechanism underlying age-related muscle loss [27,28]. Mechanistically, aging-associated alterations in nutrient sensing, reduced mTORC1 activation and impaired translational efficiency limit the ability of muscle to maintain the normal protein balance [25]. Aged muscle shows reduced mitochondrial oxidative capacity and ATP production, driven by the decline in mtDNA/mtRNA, reduced PGC-1α expression, and a ROS-mediated process that damages mitochondrial DNA and impairs fusion–fission dynamics [26,29]. In the mouse model, skeletal muscle-specific metabolic defect via the deletion of Akt1/2 directly caused accelerated sarcopenia and shortened lifespan, and phenotypes were almost reversed by additional ablation of FoxO1/4, identifying FoxO activation downstream from impaired Akt signaling as a key driver of muscle aging in mammals [30]. Concurrently, inflammaging-associated cytokine signaling engages NF-κB, JAK/STAT and p38 MAPK pathways, further favoring protein catabolism over synthesis [26]. These results indicate that anabolic resistance does not only reflect insufficient protein availability but also represents a broader failure of metabolic adaptability, in which aged muscle becomes less responsive to nutritional and mechanical cues. Therefore, therapeutic strategies targeting anabolic resistance should not only focus on increasing protein availability but also pay attention to restoring muscle responsiveness to anabolic stimuli, including adequate high-quality protein intake, timed resistance exercise, and interventions enhancing anabolic signaling. Further, as for the endocrine regulation of skeletal muscle, age-related muscle atrophy is also associated with hormonal disturbances [31]. Age-related declines in anabolic hormones and growth factors, including testosterone and circulating and muscle-local IGF-1, reduce Akt/mTOR-mediated protein synthesis, disinhibit FoxO-dependent atrophy progress, and impair satellite cell function, thereby contributing to sarcopenia [31,32]. Notably, in contrast to lower organisms, the suppression of muscle insulin/IGF-1 signaling accelerates mammalian muscle aging [30]. Testosterone stimulates muscle protein synthesis and hypertrophy through both classic androgen receptor (AR)-mediated genomic actions and the non-genomic activation of the PI3K/Akt/mTOR/S6K1 pathway [31]. In addition, testosterone promotes AR-expressing satellite cell proliferation and myonuclear accretion [31]. Testosterone also exerts anti-apoptotic effects in skeletal muscle cells [31]. However, testosterone also declines with age, a decline in total testosterone of approximately 0.8% to 1.6% per year after the age of 40 [31], resulting in the loss of skeletal muscle mass [33]. Importantly, endocrine regulation of skeletal muscle is strongly sex-dependent. This difference in circulating testosterone causes sex differences in muscle size and function [34]. Therefore, considering that many experimental studies of muscle aging have relied on male animals, these findings should be interpreted with consideration of potential sex-dependent mechanisms, particularly when extrapolating age-related pathways to females.

### 2.2. Myosteatosis

Age-related lipid deposition within and between muscle fibers (intramyocellular lipids and intermuscular adipose tissue) is termed myosteatosis [35,36,37]. Although lipid storage is an essential component of normal energy metabolism, excessive fat infiltration during aging represents a pathological remodeling process that is associated with reduced muscle quality, impaired contractile function, and metabolic dysregulation [36,38,39]. Fat infiltration has been attributed to impaired mitochondrial function in aged muscle and niche factors, causing muscle precursor cells to differentiate into adipocytes [29,37]. Therefore, myosteatosis should not only be a passive consequence of aging-associated muscle loss, but also an active pathological process about the metabolic dysfunction, impaired regeneration, and loss of muscle quality.

### 2.3. Denervation and Motor Unit Loss

Age-related muscle fiber reduction is linked to motor unit loss and fiber denervation. In older adults, neuromuscular junction (NMJ) integrity and transmission function decline [40,41]. There is also a reduction in α-motor neuron cell bodies and motor units [42,43], beginning around the age of 50 [43]. Surviving motor units initially compensate through collateral axonal sprouting and new NMJ formation, leading to enlarged motor units, increased single motor unit potential size and fiber type grouping for reinnervation [42,43,44]. However, when reinnervation becomes ineffective, muscle fibers become permanently denervated, gradually atrophy, and face inevitable death [42,43,44]. These processes underlie declines in force generation and motor control. Importantly, communication within the neuromuscular unit is bidirectional. While neural degeneration contributes to muscle atrophy, aging muscle may also negatively influence motor neuron maintenance through the reduced secretion of neurotrophic factors, like BNDF, impaired mitochondrial function, increased reactive oxygen species [45,46], and inflammaging [40,41]. Therefore, neuromuscular aging represents a self-reinforcing degenerative cycle rather than a unidirectional process. Interventions, including exercise and caloric restriction, may partially interrupt this cycle by promoting reinnervation, neurotrophic signaling, and NMJ stability [40,42,44].

### 2.4. Cellular Composition of Skeletal Muscle Tissue

Mammalian skeletal muscle is primarily composed of multinucleated, contractile muscle fibers (myocytes). It also contains mononuclear cells, including immune cells, vascular/lymphatic endothelial cells, and skeletal muscle stem/progenitor cells. Muscle is one of the few mammalian organs capable of adaptive and regenerative responses to aging. Resident muscle satellite cells (MuSCs) are crucial for regeneration, proliferating and differentiating to form new fibers [47,48]. With aging, alterations in cellular composition and function impair regenerative capacity, contributing to functional decline. The muscle niche, the microenvironment including satellite cells, immune cells, endothelial cells, neural inputs, and extracellular matrix, critically influences regeneration. Recent advances in single-cell and spatial transcriptomic technologies have transformed our understanding of aging skeletal muscle by enabling the characterization of cellular states within their native tissue contexts [49,50]. These approaches have revealed that age-related decline in muscle is not only attributable to intrinsic defects in individual cell populations but also involves disrupted intercellular communication [49,50]. Therefore, skeletal muscle aging is increasingly viewed as a complex multicellular process involving changes in diverse cellular populations and their microenvironment [50]. However, despite these advances, spatial omics approaches have not yet been systematically applied to characterize physiological skeletal muscle aging, and most current studies have focused on injury-regeneration models. In the following sections, we review the contributions of major cell types, including myofibers, muscle stem cells, fibro/adipogenic progenitors, immune cells, and vascular cells, to age-related muscle degeneration, with the caveat that spatial omics has not yet been systematically applied to physiological skeletal muscle aging.

#### 2.4.1. Myofibers

Skeletal muscle consists of fascicles (bundles of muscle cells). The cell membrane is the sarcolemma. The cytoplasm is the sarcoplasm, containing proteins, organelles, and myofibrils with actin and myosin filaments. Based on myosin heavy chain isoforms, fibers are primarily classified as: type I (slow-twitch, fatigue-resistant, high oxidative capacity), type IIA (fast-twitch, intermediate oxidative/glycolytic capacity, fatigue-resistant), and type IIB (fast-twitch, highly glycolytic) [51]. Normal aging shifts fiber distribution toward a slower profile, accompanied by endurance decrease [52]. These shifts may explain fiber type-specific susceptibility in muscle diseases. Further research is needed on mitochondrial content and transcriptional control to understand differential susceptibility of fiber types to aged-related deterioration.

#### 2.4.2. Muscle Stem Cells (Satellite Cells)

Regeneration from native precursors is crucial in maintaining functional muscle mass. Muscle stem cells, also named as muscle satellite cells (MuSCs), reside between the basal lamina and sarcolemma of the myofiber, responsible for regeneration [53]. Stem cell exhaustion is a hallmark of aging [2]. MuSCs are usually quiescent, self-renewing via asymmetric division. Muscle injury activates them. They proliferate, differentiate into myoblasts, and fuse to form new myofibers [48]. With aging, satellite cell depletion impairs tissue plasticity [26]. Walter et al. generated a lifespan-spanning single-cell and spatial transcriptomic atlas of skeletal muscle regeneration and demonstrated that aged muscles contain distinct MuSC trajectories characterized by increased senescence-associated signatures [49]. Analysis of MuSCs from aged individuals shows elevated mRNA levels of aging markers (P14, P16, P19, P21) [54]. While muscle atrophy in aging is associated with reduced satellite cell number [26], a study in geriatric sarcopenic mice found a marked drop in regenerative capacity without further reduction in satellite cell count, suggesting the decline is due to cell degeneration rather than loss [55]. For example, age-related failure to activate the Notch pathway impairs regeneration [56]. Notch signaling is essential for MuSC proliferation and differentiation. Senescent MuSCs can still regenerate if Notch is activated [57]. Intrinsic Notch and pSmad3 signaling pathways oppose each other in regeneration [58]. But their balance is disrupted by the aged microenvironment. A novel mechanism proposes that a sharp decline in fibronectin in the aging niche impairs MuSC regeneration [59]. Similarly, Kang et al. propose that molecular changes in aged muscle stem cells, such as the upregulation of N-myc downregulated gene 1 (NDRG1), may be positive compensatory responses that enhance cell survival in a harsh microenvironment, but at the expense of regenerative function [60]. They further put forward a cellular survivorship bias that during aging, muscle stem cells prioritize stem cell persistence at the expense of functionality [60]. Additionally, age-related fibrosis contributes to dysfunction. In fibrotic aged muscle, activation of canonical Wnt signaling in myogenic progenitors promotes a shift from myogenic to fibrogenic lineage, possibly due to serum components from aged mice [61]. Thus, age-related muscle degeneration may stem more from signaling dysregulation than absolute stem cell loss. Notably, MuSCs actively modulate their microenvironment [62], secreting the senescence-associated secretory phenotype (SASP), a mix of bioactive factors, to communicate with other cells [54]. Extrinsic regulation involves dynamic interactions with stromal cells [63]. Pseudotime analysis shows senescent MuSCs diverge into two transcriptomic fates, with one characterized by a profibrotic gene expression signature [54]. This branch may be a precision target for anti-fibrosis and anti-aging. Regardless of fate, about 70 SASPs are upregulated in senescent cells, and SASP-mediated intercellular interactions increase in aged muscle [54]. Satellite cells also secrete myokines, like IL-6 and IGF-1, to maintain the microenvironment. Pseudotime analysis revealed two divergent transcriptomic fates in senescent MuSCs, characterized by distinct expression patterns of SASP-related genes and potentially distinct regulatory programs. While further functional studies are required to determine whether these fates correspond to stable subpopulations, these findings suggest that senescent MuSCs exhibit functional heterogeneity in their secretory phenotypes and niche interactions. Aging impairs MuSC function via cell-autonomous mechanisms and non-cell-autonomous alterations in the cell niche. Heterochronic parabiosis in mice showed that exposure to young serum upregulated the Notch ligand Delta in old MuSCs, activating Notch and restoring proliferative and regenerative capacity [57], indicating that circulating factors in youth can reverse certain aspects of age-related decline. Consistent with this, GDF11, a circulating protein that declines with age, can reverse aged MuSC impairments when supplemented [64]. These findings establish that a portion of MuSCs, aging is reversible by circulating factors. Importantly, these rejuvenating effects of young circulating factors appear restricted to moderately aged MuSCs and to specific signaling pathways. In contrast, transplanting muscle or freshly purified satellite cells from geriatric mice into young hosts did not restore function [55], indicating that intrinsic alterations accumulate in geriatric MuSCs and are not reversed by a young environment alone. Together, these findings suggest that environmental interventions can partially ameliorate aging phenotypes in MuSCs, but are insufficient to reverse established intrinsic cellular damage. MuSC aging is best modeled as the superposition of a reversible, niche-driven component and a slowly reversible, cell-intrinsic component. Therefore, effective rejuvenation strategies will likely need to combine niche modulation with approaches that directly repair intracellular damage.

#### 2.4.3. Fibro/Adipogenic Progenitors (FAPs)

FAPs are interstitial stromal progenitors, low in proportion but essential for homeostasis and regeneration. Single-cell profiling has identified heterogeneous FAP subsets with distinct transcriptional programs, including adipogenic, pro-remodeling, and stem-like states [49]. Their proportion increases with skeletal muscle aging [54], probably indicating a shift toward abnormal differentiation (e.g., adipogenic [54,65] or pro-fibrotic [66]). A study shows primary cilia on FAPs transduce cues like Hedgehog signaling, upregulating TIMP3 (a metalloproteinase inhibitor) that prevents FAPs from becoming adipocytes [65]. In a study constructing a senescence atlas of aging human skeletal muscle using single-nucleus RNA-seq and ATAC-seq from hamstring muscle biopsies of five young (19- to 27-year-old males who underwent anterior cruciate ligament reconstruction) and five aged (60- to 77-year-old males who underwent knee replacement surgery), pseudotime trajectory analysis of FAPs indicates aged nuclei increase, senescent FAPs accumulate (defined by a unified senescence score integrating multiple senescence gene sets), cell cycle scores decrease, and inflammatory response genes are enriched in senescent FAPs [54], suggesting FAPs may contribute to tissue aging. However, a recent integrated study showed that during muscle regeneration in aged humans, FAPs communicate with monocytes/macrophages via complement C3, promoting an optimal regenerative response [67]. Further research into age-related changes in FAPs and their interactions is promising. Meanwhile, recent studies via mouse models have provided new mechanistic opinions on FAP aging. Lukjanenko and colleagues found that in the muscle of aged mice the absolute number of FAPs decreased, with the proliferative capacity impaired [68]. Additionally, the adipogenic potential of aged FAPs was reduced compared to young controls, whereas the fibrogenic differentiation was enhanced [68]. Notably, transplantation of young FAPs rescued the abundance of MuSCs in aged mice [68]. Future studies on age-related changes in FAPs and their interactions with MuSCs and other cells hold promise for developing new strategies to delay skeletal muscle aging.

#### 2.4.4. Immune Cells

Age-related immune infiltration may mediate the pro-inflammatory environment of aged muscle [69]. Walter et al. identified eight immune cell populations exhibiting age-dependent alterations during muscle regeneration, demonstrating that immune responses change with aging [49]. Among the various immune cells in skeletal muscle, macrophages play a prominent role in aging-associated inflammation and regeneration [70]. Here, we discuss macrophages as a key example of immune cell involvement in age-related muscle dysfunction. Effective muscle regeneration post-injury requires an orchestrated inflammatory response with regulated immune cell infiltration and cytokine production [71]. Upon injury, quiescent satellite cells activate and interact with infiltrating immune cells to initiate proliferation and differentiation [43,57]. A moderate inflammatory response clears debris and initiates regeneration [72]. However, persistent or excessive inflammation inhibits regeneration and promotes atrophy [73]. This is tied to the timely switch from a pro-inflammatory to an anti-inflammatory milieu, mirroring macrophage polarization from M1 to M2 [74]. Early pro-inflammatory M1 macrophages facilitate myoblast expansion and later, M2 macrophages promote myogenic cell differentiation and fusion into new myotubes [75]. Thus, macrophage polarization regulates regeneration, and the M1/M2 balance is critical for resolving inflammation and restoring function. This balance is disrupted in aging muscle [76]. Senescent tissue secretes SASPs, upregulating pro-inflammatory molecules (CXCL, Visfatin, ALCAM), exacerbating macrophage infiltration and disrupting niche function [54]. Aged muscle also shows increased levels of 15-PGDH, a prostaglandin E2 (PGE2)-degrading enzyme expressed in fibers and interstitial cells like macrophages [77]. The subsequent decline in PGE2 signaling contributes to atrophy.

#### 2.4.5. Endothelial Cells

Capillary density and endothelial function decline with age, limiting muscle perfusion. Recent evidence indicates that aged endothelial cells of skeletal muscle exhibit age-related transcriptional changes [78]. The diminished proliferative capacity of endothelial cells contributes to impaired angiogenesis in aging, which is driven by the alterations in NAD+-dependent metabolism [78]. Exercise-induced angiogenesis in skeletal muscle is partly regulated by PGC-1α-mediated VEGF signaling [79,80]. Additionally, the NAD+-dependent deacetylase SIRT1 has been shown to be an intrinsic negative modulator of Notch signaling in endothelial cells [81], and supplementation of NAD+ precursor NMN restores the activity of SIRT1, thereby improving age-associated vascular dysfunction and reducing oxidative stress in mice [82]. Additionally, human aging atlases further demonstrate that vascular-associated cells participate in broader age-related remodeling of the skeletal muscle ecosystem, potentially influencing nutrient delivery, inflammatory regulation, and stem cell niche maintenance [69].

#### 2.4.6. Senescent Cells

Senescent cells are a major niche component, influencing regenerative capacity. Cellular senescence is an adaptive response to stressors, leading to irreversible cell cycle arrest [83]. Critically, senescent cells (SnCs) secrete SASPs (pro-inflammatory cytokines, chemokines, growth factors, matrix-remodeling enzymes) [54]. SnCs disrupt tissue homeostasis via SASPs’ paracrine action, promoting chronic inflammation, fibrosis, and progenitor dysfunction [54]. SASPs can directly or indirectly foster inflammation [54]. Intricate interactions exist among aged muscle cell populations, with SASPs playing a pivotal role. Transcription factors NF-κB and C/EBPβ are key regulators of senescence and SASPs [84,85]. Current anti-aging strategies often focus on eliminating SnCs or neutralizing SASPs. Recently, the AP-1 family has emerged as a potential regulator of muscle senescence/aging [54], representing a promising target for senomorphic interventions.

## 3. Skeletal Muscle Orchestrates Organismal Metabolic Homeostasis and Aging

As a highly plastic, major endocrine organ, aged skeletal muscle is not only a passive responder to systemic aging with the loss of mass and strength, but also an active driver that modulates systemic metabolic dysfunction, inflammation and immune status via myokine secretion, influencing age-related diseases and overall aging. Several studies in mammals and fruit flies demonstrated that stress-sensing in skeletal muscle influenced systemic aging and lifespan, suggesting that myokines between aged skeletal muscle and the whole body may play a key role in the aging process [22]. The major age-related changes in muscle-derived myokines and their biological effects are summarized in Table 1. In addition, an increasing number of studies show that muscle-specific clocks can predict systemic aging.

### 3.1. Glucose Homeostasis

Skeletal muscle is a primary insulin-sensitive tissue crucial for systemic glucose homeostasis [100]. GLUT4 mediates glucose transport into muscle [100]. Muscle-specific GLUT4 knockout in mice causes severe insulin resistance and impaired glucose tolerance, underscoring its essential role [100]. However, with advancing age, skeletal muscle GLUT-4 protein concentration decreases and may at least partially contribute to the insulin resistance [101]. Fortunately, exercise training increases skeletal muscle GLUT-4 protein concentration and thereby enhances systemic insulin sensitivity, occurring in both older and younger individuals [102].

Myokines secreted by muscle can modulate systemic glucose metabolism and change with age. Irisin, a peptide produced by muscle during exercise, promotes white adipose tissue browning by upregulating UCP-1, enhances muscle glucose metabolism, reduces visceral fat, and improves insulin sensitivity [86,103,104,105]. Circulating irisin declines with age and may contribute to visceral fat accumulation and non-alcoholic fatty liver disease progression, which exercise reverses to ameliorate insulin resistance [106,107]. FGF21, secreted by muscle in adaptive responses to mitochondrial stress, elevates metabolic rate and improves systemic insulin sensitivity when circulating levels are high [89]. Muscle-specific loss of FGF21 completely obliterates the metabolic protective effects, leading to the resurgence of diet-induced obesity and insulin resistance in mice [89]. Meanwhile, in age-related chronic mitochondrial dysfunction, compensatory FGF21 upregulation often becomes insufficient, failing to prevent pathological progression [90]. Similar to FGF21, recent studies have identified another myokine, Growth Differentiation Factor 15, GDF15, is also a mitochondrial stress-associated myokine, secreted by muscle, and improves systemic metabolism by promoting lipolysis and fatty acid oxidation [95]. A study found that GDF15 expression was increased in skeletal muscle rather than liver, adipose tissue, kidney or heart of middle-aged mice [92]. GDF15 expression increases with aging and associates with the loss of muscle mass [93]. In a mouse model with the knockout of taurine transporter that exhibits accelerated skeletal muscle aging, GDF15 expression was markedly higher and circulating levels were also elevated, which may control aging phenotype [94]. Related changes by GDF15 remain to be fully elucidated. Moreover, IL-6 exerted opposing effects on systemic homeostasis. In young, IL-6 secreted by exercised muscle enhances muscle glucose uptake, lipolysis, and fatty acid oxidation via AMPK [108]. Via STAT3 phosphorylation, it also activates UCP-1 and PGC-1α, promoting white adipose browning and further alleviating insulin resistance [109]. With aging, however, IL-6 switches to a pro-inflammatory profile, contributing to systemic low-grade inflammation and exacerbating insulin resistance [110]. IL-6-deficient mice have increased adiposity, partially reversed by IL-6 treatment [111]. Collectively, the age-related decline in protective myokines, like irisin, and the emergence of a pro-inflammatory myokine profile, like IL-6, transforms skeletal muscle from a guardian of glucose homeostasis into a driver of systemic metabolic dysfunction.

### 3.2. Skeletal Muscle Thermogenesis and Energy Homeostasis

Beyond the role in locomotion and endocrine regulation, skeletal muscle represents a major thermogenic organ that contributes to the whole-body energy expenditure and metabolic homeostasis. Skeletal muscle thermogenesis includes shivering thermogenesis mediated by ATP-consuming muscle contraction and non-shivering thermogenesis involving sarcoplasmic reticulum calcium cycling [112]. In adult humans, brown adipose tissue, BAT, contributes less than 1% to whole-body thermogenesis even under cold exposure, whereas skeletal muscle accounts for about 50% of cold-induced energy expenditure [113]. With aging, the thermogenic capacity of BAT declines, and skeletal muscle undergoes a compensatory shift for greater thermogenesis [114]. However, the compensatory adaptation of aged muscle contributes to energetic stress and oxidative damage, which may result in sarcopenia [114]. Recently, sarcopenia is increasingly recognized as a condition characterized by dysregulation of systemic energy metabolism, rather than merely an imbalance between muscle protein synthesis and breakdown [114].

Interorgan metabolic networks are also involved. When skeletal muscle thermogenesis is restricted, BAT is activated for thermogenesis, accompanied by enhanced white adipose tissue lipolysis and hepatic gluconeogenesis [115]. Evidence in mice indicated that IL-6 mediated branched-chain amino acid mobilization from skeletal muscle to BAT for thermogenesis [115]. The relevance of this axis to human aging requires further investigation, as BAT contributes less to the whole-body thermogenesis in adult humans [113]. Skeletal muscle supplies amino acids to the liver for gluconeogenesis via the Cori cycle and glucose–alanine cycle for thermogenesis [116,117,118]. This interorgan exchange has been demonstrated in mice [117] and sheep [118]. The gut microbiota modulated muscle metabolism through short-chain fatty acids and short-chain fatty acids and hormones, including glucagon-like peptide-1 (GLP-1) and glucose-dependent insulinotropic polypeptide (GIP) [119,120]. And in mice, temperature fluctuations aggravated muscle atrophy via gut microbiota alterations [121]. Muscle-derived myokines such as IL-6 and irisin have involved in adipose browning [87]. Additionally, the muscle-enriched myokine Musclin binds TfR1, antagonizing thermogenesis via the TfR1-mediated cAMP/PKA pathway, suppressing brown adipocyte activity and exacerbating obesity-related metabolic disorders [99]. Taken together, with aging, the diminished thermogenesis of muscle and BAT has been regarded as being associated with decreased BMR and muscle mass. A vicious cycle may therefore arise, in which age-related muscle loss impairs thermogenic capacity, and the thermoregulatory metabolic burden of skeletal muscle increases, further exacerbating atrophy [114].

### 3.3. Bone and Muscle Crosstalk in Aging

Bone and skeletal muscle are interconnected structurally and functionally through mechanical loading and endocrine communication. This bone–muscle axis is mediated by myokines, osteokines and extracellular vesicles, which coordinate bone remodeling, muscle regeneration, and systemic metabolism [122,123,124,125,126,127]. In mice, aerobic exercise enhanced endurance capacity by restoring muscle glycogen content, whereas osteocalcin neutralization abolished this effect [127]. Beyond its role in exercise-induced muscle adaptation, studies using isolated mouse extensor digitorum longus and soleus muscles showed that undercarboxylated osteocalcin directly stimulated glucose uptake in the absence of insulin [128], and restored impaired insulin-stimulated glucose uptake in isolated muscles from corticosterone-treated mice, particularly in extensor digitorum longus muscle [129]. In human observational studies, total osteocalcin concentrations in serum declined from relatively high levels in young adulthood in both sexes [130]. In women, concentrations reached its lowest level at approximately 35–39 years of age before increasing around the menopausal transition, whereas in men, they declined more gradually until approximately 60–70 years of age [131], suggesting that osteocalcin declined early in adulthood, and reduced earlier in female possibly resulting from the difference of sexual hormones between female and male. Exercise-induced IL-6 in muscle promoted osteoclast differentiation, with the consequent release of the osteocalcin [132]. However, these human findings are observational and the mechanisms remain to be proven. Furthermore, muscle-derived CLCF1 inhibited osteoclastogenesis via STAT1 and promoted osteoblastogenesis via STAT3, ameliorating age-related muscle weakness and bone loss [124]. Blocking CLCF1 abolished the benefits of exercise in mice [124]. Therefore, CLCF1 represented a muscle-derived endocrine mediator linking exercise-induced muscle adaptation with bone remodeling. Extracellular vesicles are cell-derived, non-nucleated, non-replicating, membrane-delimited particles that transport molecular cargo through the circulation and regulate distant target cells [122]. Skeletal muscle-derived extracellular vesicle-miRNAs induced osteogenesis, which changed with age and contributed to osteosarcopenia [122,126]. Bone-derived extracellular vesicle-miRNAs were also remodeled with age, which may shift bone–muscle communication toward a catabolic state [122]. Therapeutics that restore the bone and muscle communication could enhance healthspan. Moreover, the age-related disruption of the bone–muscle axis is aggravated by the systemic aging processes. The expansion of bone marrow adipose tissue and intermuscular adipose tissue is linked to increased secretion of pro-inflammatory adipokines, impairing the differentiation and survival of muscle and bone precursors and contributing to the pathophysiology of osteoporosis, sarcopenia and osteosarcopenia [122]. Cellular senescence and SASPs enhance osteoclastogenesis, inhibit myogenesis, and impair tissue regeneration [123]. Therefore, osteosarcopenia represents not only the coexistence of osteoporosis and sarcopenia but also a consequence of disrupted interorgan communication during aging.

### 3.4. Cardiovascular Regulation

Multiple factors related to muscle secretions protect the cardiovascular system. GDF15 exhibits cardioprotective properties through resisting pressure overload-induced hypertrophy [96], protecting cultured cardiomyocytes from apoptosis during stimulated ischemia/reperfusion [97] and suppressing leukocyte integrin activation, by which myocardial infarction mice were enabled to survive [98]. On the contrary, the age-related increase in GDF15, also emerging as a cardiovascular risk factor, is significantly associated with arterial stiffness and all-cause mortality [133]. Myonectin (CTRP15/erythroferrone), an exercise-induced myokine, inhibits apoptosis and inflammation in the heart during acute myocardial ischemia, mediating the cardiovascular benefits of exercise [134]. Lactate, the most abundant myokine [88], influences metabolic regulation by activating SIRT1/3 and modulating redox status [135]. In exercise training, lactate upregulates angiogenesis factors like VEGF [136]. Lactate also provides energy for the heart and regulates cardiac output during exercise [135].

In conclusion, some myokines have cardioprotective effects and may change with age, contributing to the increased cardiovascular vulnerability observed with advancing age.

### 3.5. Muscle–Brain Communication

Skeletal muscle secretes myokines to maintain proteostasis in target tissues like the brain and retina [137]. Proteasome dysfunction is a hallmark of aging [2]. Clinical observational studies suggest muscle is a key regulator in brain and retinal aging via myokines [137]. Research in aged Drosophila shows that under mild proteostatic stress, muscle secretes the amylase Amyrel [137]. A novel endocrine stress axis, muscle-derived amylase and the CNS, is proposed [137]. Amyrel generates maltose, a central proteostasis signaling molecule, activating chaperones/proteases and reducing protein aggregation in aged mouse and fly brain/retina, preserving proteostasis and delaying neurodegeneration [137]. Validation in human brain organoids showed maltose supplementation under heat stress improved proteostasis and protected neurons [137]. Targeting the maltose pathway may be a novel therapeutic strategy for conditions like Alzheimer’s disease and age-related macular degeneration. Also, BDNF, released by contracting muscle in a calcium-dependent manner, maintains satellite cell proliferation/differentiation and neuromuscular junction development [138]. A study of male mice demonstrated that lactate crossed the blood–brain barrier, increased BDNF, promoted hippocampal neurogenesis, and improved cognition [139]. Studies in young men showed that exercise-induced increases in lactate [140] or intravascular lactate infusion [141] stimulated BDNF release, suggesting that lactate was an important molecule for exercise-induced increase in BDNF and promoted cognitive function.

However, previous studies have largely relied on animal models, lacking studies on the impact of muscle molecular age on systemic aging in humans. In recent years, the development of molecular aging clocks has provided novel tools to quantify the relationship between muscle and systemic aging in humans. A study showed that muscle-related proteins in blood reflected systemic aging in advance and the acceleration of the muscle proteomic clock independently predicted mortality [142]. The muscle proteomic clock was defined as the difference between an individual’s muscle-specific biological age, which is estimated from muscle-related proteins in blood by a machine learning model, and their chronological age [142]. However, this is an observational study, the causal direction between skeletal muscle aging and systemic aging cannot be determined. To investigate whether muscle molecular aging directly drives systemic aging, a recent study used an adeno-associated virus, a gene delivery tool, to restore expression of Bmal1, a core circadian transcription factor, specifically in the skeletal muscle of whole-body Bmal1 knockout mice [143]. The work showed that this muscle-specific restoration of the factor significantly extended lifespan, improved motor performance and glucose metabolism, and reduced systemic inflammation, independent of changes in muscle mass [143]. This firstly and directly demonstrated that the circadian clock in skeletal muscle causally regulated systemic health and longevity, suggesting that the improvement in muscle homeostasis had a positive effect on systemic health.

In summary, skeletal muscle modulates systemic aging and age-related disease progression by secreting diverse soluble factors in response to internal and external cues. Skeletal muscle not only mediates tissue crosstalk via the classical secretory factors described above, but also through extracellular vesicles during exercise, rapidly releasing a large number of proteins into the circulation for tissue crosstalk [144]. Underlying secretory mechanisms remain to be fully elucidated. However, notably, age-related loss of muscle mass and function reduces physical activity and may impair the production and function of key exercise-induced myokines, thereby diminishing the benefits of exercise and potentially accelerating age-related degenerative changes.

## 4. Targeting Skeletal Muscle to Ameliorate Systemic Aging

**Figure 2:**
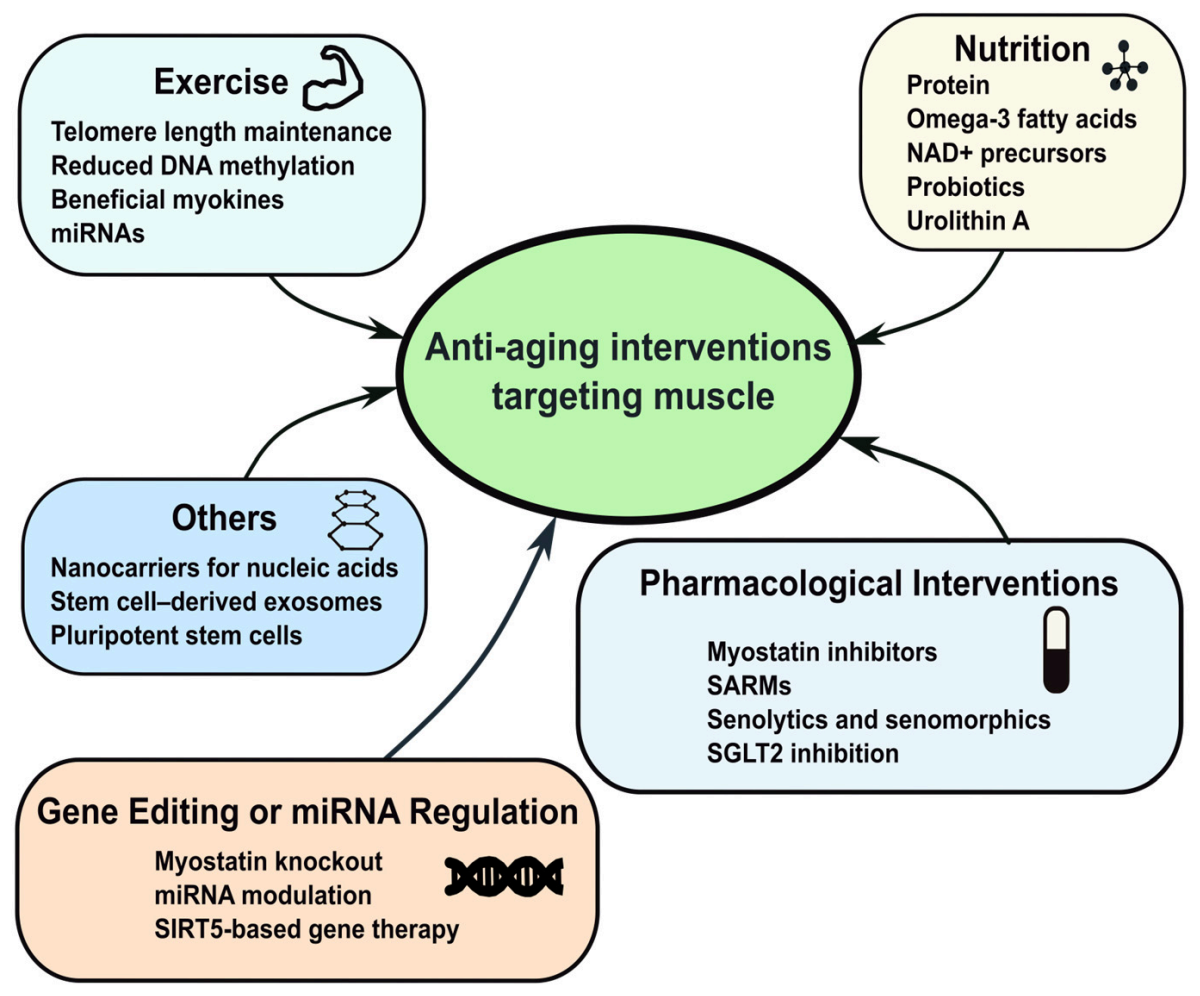
A framework of several anti-aging interventions targeting muscle. This framework displays the importance of exercise, nutrition, pharmacological interventions, gene editing or miRNA regulation, and the medical applications of nanomaterials for ameliorating muscle aging. Created with Inkscape version 1.3.2 (091e20e, 25 November 2023, custom).

Given the crucial role of skeletal muscle, anti-aging interventions targeting this tissue may help ameliorate systemic aging, as shown in Figure 2 below.

### 4.1. Exercise

Resistance and aerobic training robustly improve muscle mass, strength, metabolic health, and trigger beneficial myokine release [86,88,108,134]. Quantitative evidence indicates that exercise can partially attenuate molecular hallmarks of aging. In aged mice, 8 weeks of combined endurance and resistance exercise reduced skeletal muscle epigenetic age by approximately 8 weeks, a 10% relative reduction compared with aged sedentary controls [145]. In older adults, 12 weeks of explosive-type resistance training preserved leukocyte telomere length, with a post-intervention telomere repeat copy number to single-copy gene copy number (T/S) ratio of 5.8 ± 0.3 in the training group versus 3.9 ± 0.2 in controls, and reduced global DNA methylation by approximately 18–20% [146]. Exercise modulates epigenetic marks and telomere dynamics, attenuating epigenetic aging and preserving function even when initiated later in life [145,146]. Telomere attrition and epigenetic alterations are aging hallmarks [2]. A study in older adults with regular resistance training found it associated with better leukocyte telomere length maintenance and reduced global DNA methylation [146]. Telomere length correlated positively with functional power and strength tests [146]. Exercise alters DNA methylation and transcription of genes related to muscle structure, metabolism, and mitochondrial function [147]. Exercise-induced ROS shapes the epigenome for muscular adaptation [148]. Exercise may influence methylation of aging-related genes by improving redox homeostasis, delaying aging phenotypes [149]. Even late-life exercise can attenuate skeletal muscle epigenetic aging [145]. Compared to younger individuals, older adults exhibit more pronounced changes in DNA methylation following exercise, possibly because the accumulation of epigenetic errors is related to age, which may be counteracted by exercise to reverse these errors, offering a degree of protection against the age-related diseases and frailty [150]. Additionally, exercise changes the expression of miRNAs (e.g., miR-1, miR-133), which regulate genes involved in myogenesis and metabolism [151], highlighting the molecular mechanisms of miRNAs in skeletal muscle development [152]. Exercise is now recognized as a potent trigger for the systemic release of extracellular vesicles (EVs) into circulation. These EVs together with other circulating EVs serve as efficient carriers of protected bioactive cargo, including proteins, mRNAs, and miRNAs, which target distal tissues such as the liver and brain [144,153,154]. Specifically, exercise-derived EVs have been shown to stimulate mitochondrial biogenesis via the PGC-1α pathway and to enhance adult hippocampal neurogenesis in vivo, constituting a novel and powerful mechanism for beneficial muscle–brain and muscle–organ crosstalk [154,155]. Importantly, aging severely compromises this protective EV signaling network by shifting EV subpopulation heterogeneity and cargo composition, resulting in elevated pro-aging miRNAs (e.g., miR-29a-3p, miR-34a-5p) and reduced longevity factors (e.g., Klotho mRNA) [153,154].

### 4.2. Nutrition

Adequate high-quality protein, omega-3 fatty acids, and targeted probiotics may support muscle protein synthesis and function maintenance. Adequate protein intake can mitigate age-related anabolic resistance and preserve muscle mass, which in turn reduces frailty-associated mortality [156]. However, unlike exercise interventions, current evidence does not yet demonstrate measurable reversal of epigenetic age or preservation of telomere length following protein supplementation. Creatine supplementation presents a muscle-targeted intervention that may attenuate the effects of skeletal muscle aging. Studies indicate that creatine, particularly when combined with resistance training, improves lean mass, muscle strength and physical performance in older adults [157]. Omega-3s are anti-inflammatory and enhance protein synthetic response [158]. Supplementation with omega-3 fatty acids enhances the anabolic response of skeletal muscle. In older adults, 8 weeks of fish oil supplementation, which provided 1.86 g/day eicosapentaenoic acid and 1.50 g/day docosahexaenoic acid, increased muscle protein synthetic response by approximately 40% [158]. Moreover, a study showed that in aged human skeletal muscle, NAD^+^ levels are markedly reduced and positively correlate with muscle function [159]. A randomized controlled trial in postmenopausal women with prediabetes showed that NMN supplementation enhanced skeletal muscle insulin sensitivity [160]. NAD^+^ deficiency is a core feature of sarcopenia, and NAD^+^ precursor supplementation may hold therapeutic potential [161]. Trigonelline, one of the NAD^+^ precursors, can improve muscle function during aging. Dietary supplementation of trigonelline in male mice enhances muscle strength and prevents fatigue during aging [162]. Twelve weeks of dietary trigonelline supplementation increased grip strength by about 14% in aged mice [162]. Serum trigonelline levels are reduced in human sarcopenia and positively correlate with grip strength and muscle mass in humans, suggesting that trigonelline may have therapeutic potential for human muscle aging [162]. Importantly, emerging evidence suggests that the biological actions of trigonelline may extend beyond its proposed role as an NAD^+^ precursor. In a mouse model of skeletal muscle ischemia–reperfusion injury, trigonelline improved mitochondrial calcium handling and reduced calpain activity, accompanied by increased histological evidence of muscle fiber regeneration [163]. However, because this study did not directly measure NAD^+^ levels [163], it remains unclear whether these effects are mediated by NAD^+^-dependent mechanisms or involve additional pathways. Furthermore, nicotinamide and pyridoxine, both generally recognized as safe at nutritional doses, may act synergistically to promote the proliferation and differentiation of MuSCs, accelerate post-injury regeneration and counteract age-related stem cell exhaustion [164]. Probiotics like Lactobacillus casei strain Shirota [165] produce short-chain fatty acids modulating the gut–muscle axis, improving muscle inflammation. Similarly, urolithin A, a postbiotic metabolite generated and produced by gut microbiota from ellagic acid, represents a novel nutritional intervention targeting mitochondrial quality control in skeletal muscle aging [166]. Recent randomized controlled trials have provided translational evidence supporting its potential benefits. In middle-aged adults, four months of UA supplementation improved muscle strength, exercise performance, and circulating biomarkers related to mitochondrial health [166]. Consistently, in older individuals aged 65 to 90 years, four months of UA supplementation increased muscle endurance compared with placebo, supporting its potential role in mitigating age-related decline in muscle function [167].

### 4.3. Pharmacological Interventions

Myostatin, a member of the TGF-β superfamily and a negative regulator of skeletal muscle mass, has emerged as a target for combating age-associated muscle loss [91,168,169]. Myostatin antibody has been evaluated as a therapeutic strategy for increasing lean muscle mass in a phase-2 trial of older adults [170]. However, despite meeting its primary endpoint, the anti-myostatin antibody LY2495655 (landogrozumab) ultimately failed in later development. Additionally, bimagrumab, an inhibitor of the myostatin/ActRII signaling pathway, was evaluated in a randomized clinical trial involving 180 community-dwelling adults (mean age 79.1 ± 5.3 years) [171]. Twenty-four weeks of treatment with bimagrumab increased lean body mass by 7% compared with 1% in the placebo group [171]. However, despite the increase in lean body mass, no significant improvements were observed in physical performance [171].

Selective androgen receptor modulators (SARMs) represent another anabolic strategy through activating androgen receptor signaling [172]. Enobosarm, one of the most extensively studied SARMs, was developed for age-related muscle loss and cancer cachexia [173]. Early phase-II clinical trials demonstrated dose-dependent increases in lean body mass and improvements in stair-climbing power in healthy older adults and postmenopausal women [173]. However, similar to bimagrumab, although enobosarm increased lean body mass, it failed to complete functional endpoints [174]. Recently, enobosarm has been reconsidered as a promising strategy for the preservation of lean mass during GLP-1 receptor agonist [175].

Taurine, a semi-essential micronutrient, has emerged as a potential regulator of biological aging [176]. A study finds that serum concentrations of taurine decline with aging in mice, monkeys, and humans [176]. Supplementation with taurine has been reported to increase the healthy lifespan in worms and mice, and healthspan in monkeys via reducing cellular senescence, suppressing mitochondrial dysfunction and attenuating inflammaging [176]. However, although preclinical evidence is encouraging, human clinical trials are required to determine whether the decrease in taurine drives human aging.

The accumulation of senescent cells within the muscle microenvironment can impair tissue regeneration and promote chronic inflammation through the secretion of SASPs. Senolytics and senomorphics that clear or modulate senescent cells/SASPs are emerging strategies to restore niche function and improve regeneration [177]. In a murine model of radiation-induced senescence, treatment with the senolytic combination dasatinib and quercetin reduced the expression of senescent markers and improved muscle function [178]. Similarly, in a rat model of Duchenne muscular dystrophy, a progressive disease characterized by chronic muscle degeneration and inflammation, senolytic intervention has also proven effective as it attenuated the loss of body weight and muscle strength while promoting muscle regeneration, even at late stages of the disease [179]. Notably, current evidence for senolytic therapies in skeletal muscle aging remains predominantly derived from preclinical models, and clinical studies specifically evaluating their effects on age-associated muscle dysfunction or sarcopenia are still limited. And exercise has emerged as a potential endogenous senolytic/senomorphic intervention that may reduce senescence-associated alterations and improve muscle health during aging [180]. A recent study demonstrated that SGLT2 inhibition promoted the in vivo clearance of senescent cells and ameliorated age-associated pathological phenotypes in mice [181].

### 4.4. Gene Editing or miRNA Regulation

Gene-editing approaches (e.g., myostatin knockout) and miRNA modulation can provide opportunities to modify pathways regulating muscle aging and increase muscle mass and influence MuSC function in models [182,183]. Recent primate research revealed that SIRT5 delays muscle aging by promoting TBK1 desuccinylation to inhibit pro-inflammatory signaling. A SIRT5-based gene therapy achieved rejuvenation of aged muscle in vivo [184]. MicroRNAs (miRNAs) are important post-transcriptional regulators of skeletal muscle homeostasis [185]. Aging alters the expression profile of muscle-derived miRNAs, affecting muscle function and regeneration [185,186]. Accordingly, modulation of aging-associated miRNAs represents a potential therapeutic strategy for delaying muscle aging, which lies in the ability of miRNAs to act as molecular switches [187]. However, miRNA modulation remains largely preclinical and requires validation in human trials as muscle-specific delivery strategies.

### 4.5. Others

Advanced delivery and regenerative strategies of nanocarriers for nucleic acids, stem cell-derived exosomes, and induced pluripotent stem cells present potential therapeutic tools for improving muscle regeneration [188,189].

Limitations and Future Perspectives

Collectively, these advances provide new insights into the mechanisms underlying skeletal muscle aging and potential therapeutic opportunities for preserving muscle mass and function. However, differences in lifespan, muscle fiber composition, regenerative capacity, and systemic aging processes should be considered when interpreting experimental findings. Future studies that integrate multi-omics approaches across species and incorporate human longitudinal cohorts and clinical trials will be essential to bridge mechanistic discoveries and effective interventions for age-related muscle dysfunction. Importantly, not all age-related alterations in skeletal muscle are equally reversible. Several metabolic alterations are highly modifiable. Exercise can partially restore age-related mitochondrial defects and preserved mitochondrial plasticity even in later life [190]. Additionally, resistance exercise remains effective in improving muscle mass and function in older adults [145]. Nutritional interventions, such as protein, omega-3 fatty acids, creatine, and so on, may support anabolic signaling, mitochondrial function, and inflammatory regulation. Cellular senescence represents another potentially reversible contributor to muscle aging. Senolytic treatment with dasatinib and quercetin reduced senescent cell burden and improved hypertrophic responses in aged mice, suggesting that the aged muscle microenvironment remains modifiable [191]. However, clinical translation of senolytic strategies remains limited because current evidence is mainly derived from preclinical studies [191]. In contrast, some structural alterations show limited reversibility. Although exercise may preserve neuromuscular junction (NMJ) integrity, it is unlikely to fully restore the lost motor units [192]. Therefore, therapeutic strategies should distinguish between reversible dysfunction and irreversible structural decline. Exercise, nutrition, pharmacological interventions, gene-based approaches, and regenerative therapies may target modifiable processes including mitochondrial dysfunction, anabolic resistance, epigenetic alterations, and cellular senescence. However, replacement of lost motor units or restoration of severe structural damage will require advanced regenerative strategies rather than lifestyle interventions alone. Therefore, future interventions should distinguish between restoring impaired regulatory pathways and attempting to replace irreversible cellular loss.

## 5. Conclusions

Skeletal muscle aging is a multifaceted process involving the intrinsic deterioration of muscle homeostasis and altered interorgan communication through endocrine and metabolic factors that contribute to systemic aging. Skeletal muscle influences organismal aging through myokines and metabolites, extracellular vesicles, thermogenesis, and bidirectional communication with the brain, bone, cardiovascular system, and metabolic organs. Importantly, recent single-cell and multi-omic atlases refine understanding of cell-type-specific senescence, niche dysregulation, and intercellular signaling that drives impaired regeneration and systemic decline, which hold promise for improving understanding of the causal relationships linking muscle aging to systemic aging. Integrated interventions combining exercise, optimized nutrition, targeted pharmacology, senotherapeutics (therapies targeting cellular senescence), and regenerative medicine hold the greatest promise to preserve muscle health and extend healthy lifespan.

## Figures and Tables

**Table 1:** Age-associated changes in skeletal muscle-derived myokines and their systemic effects. Aging is characterized by reduced secretion of anabolic and regenerative myokines and increased expression of catabolic or inflammatory myokines, resulting in impaired muscle homeostasis and altered communication with distant organs.

Myokine	Age-Related Alteration	Effect on Muscle Aging	Crosstalk with Other Organs	Reference
Irisin	Decreases in older adults	Improves muscle regeneration	Promotes white adipose tissue browning, increases energy expenditure and improves insulin sensitivity	[16,86,87]
BDNF	Decreases with aging	Maintains neuromuscular junction integrity, promotes muscle regeneration and counters pathological muscle fiber-type shifts	Mediates muscle–brain communication and neuroprotection of exercise	[16,40,41,45,46,88]
FGF21	Increases compensatorily in age-related chronic mitochondrial dysfunction	Acts as a stress-responsive myokine linking mitochondrial dysfunction to muscle and systemic responses	Regulates systemic glucose, lipid and energy homeostasis, and irisin-induced beige adipogenesis	[16,87,89,90]
Myostatin	Decreases with age	Inhibits skeletal muscle hypertrophy and promotes muscle atrophy and impaired regeneration	Negatively regulates white adipose tissue browning and is involved in adipose tissue function and mass regulation	[32,87,91]
IL-6	Context-dependent: transient exercise-induced increase and chronic aging-associated increase	Exercise-associated IL-6 supports metabolic adaptation whereas chronic systemic elevation is described as pathological in inflammaging	Regulates adipose tissue lipolysis, glucose metabolism and immune responses	[16,87]
GDF15	Increases during mitochondrial stress and aging	Reflects mitochondrial stress response and excessive elevation associates with muscle loss	Improves systemic metabolism by promoting lipolysis and fatty acid oxidation; exhibits cardioprotective effects by attenuating pressure overload-induced cardiac hypertrophy, reducing ischemia/reperfusion-induced cardiomyocyte apoptosis, and suppressing leukocyte integrin activation; age-related elevation is significantly associated with arterial stiffness and all-cause mortality	[92,93,94,95,96,97,98]
Musclin	Increases in obesity and temperature-associated changes	Impairs insulin sensitivity of skeletal muscle	Suppresses beige adipose tissue thermogenesis and exacerbates obesity-associated metabolic dysfunction	[16,87,99]

## Data Availability

No new data were created or analyzed in this study. Data sharing is not applicable to this article.

## Abbreviations

The following abbreviations are used in this manuscript:

15-PGDH	15-hydroxyprostaglandin dehydrogenase, the key enzyme that degrades prostaglandin E2 (PGE2)
ALCAM	Activated leukocyte cell adhesion molecule, an immunoglobulin involved in cell adhesion and signaling
AMPK	AMP-activated protein kinase, a cellular energy sensor and metabolic regulator
AP-1	Activator protein 1, a family of transcription factors involved in proliferation and stress response
BDNF	Brain-derived neurotrophic factor, a growth factor important for neuronal survival and plasticity
cAMP	Cyclic adenosine monophosphate, a key second messenger
C/EBPβ	CCAAT/enhancer-binding protein beta, a transcription factor involved in differentiation and inflammation
CNS	Central nervous system, comprising the brain and spinal cord
CTRP15	A myokine, also known as C1q/TNF-related protein 15, involved in lipid metabolism
CXCL	C-X-C motif chemokine ligand, a family of chemotactic cytokines
DNA	Deoxyribonucleic acid, the molecule that carries genetic instructions
FAPs	Fibro-adipogenic progenitors, interstitial cells in muscle that can become fibroblasts or adipocytes
GDF11	Growth differentiation factor 11, a circulating factor in aging research
GLP-1	Glucagon-like peptide-1, an incretin hormone that stimulates insulin secretion
GLUT4	Glucose transporter type 4, the insulin-responsive glucose transporter
IGF-1	Insulin-like growth factor 1, a hormone important for growth and anabolism
IL-6	Interleukin-6, a cytokine with roles in inflammation and metabolism
M1	Classically activated macrophage, a pro-inflammatory immune cell type
M2	Alternatively activated macrophage, an anti-inflammatory/repair immune cell type
MPB	Muscle protein breakdown, a process of degrading existing muscle proteins
MPS	Muscle protein synthesis, a process of building new muscle proteins
MuSC	Muscle satellite cell, a muscle stem cell responsible for growth and repair
NAD^+^	Nicotinamide adenine dinucleotide, a crucial coenzyme in redox reactions and signaling
NF-κB	Nuclear factor kappa-light-chain-enhancer of activated B cells, a key pro-inflammatory transcription factor
NMN	Nicotinamide mononucleotide, a precursor for NAD^+^ biosynthesis
PGC-1α	Peroxisome proliferator-activated receptor gamma coactivator 1-alpha, a master regulator of mitochondrial biogenesis
PGE2	Prostaglandin E2, a key lipid mediator involved in inflammation, pain, fever, and muscle metabolism and regeneration.
PKA	Protein kinase A, a cAMP-dependent kinase that phosphorylates target proteins
ROS	Reactive oxygen species, chemically reactive molecules containing oxygen
SASP	Senescence-associated secretory phenotype, a secretome from senescent cells
SIRT1	Sirtuin 1, an NAD^+^-dependent deacetylase regulating metabolism and stress response
SIRT5	Sirtuin 5, an NAD^+^-dependent deacylase regulating mitochondrial function and metabolism
SnCs	Senescent cells, cells that have irreversibly stopped dividing and exhibit the SASP
STAT3	Signal transducer and activator of transcription 3, a transcription factor in cytokine signaling
TBK1	TANK-binding kinase 1, a kinase involved in inflammatory signaling and autophagy
TfR1	Transferrin receptor 1, the main receptor for cellular iron uptake
UCP-1	Uncoupling protein 1, a mitochondrial protein for non-shivering thermogenesis in brown fat
VEGF	Vascular endothelial growth factor, a key signaling protein for angiogenesis
Visfatin	An adipokine with insulin-mimetic and pro-inflammatory properties
